# Integrative Expression and Prognosis Analysis of DHX37 in Human Cancers by Data Mining

**DOI:** 10.1155/2021/6576210

**Published:** 2021-01-02

**Authors:** Kang Huang, Tudi Pang, Changjun Tong, Houqing Chen, Yupeng Nie, Jiayi Wu, Yandong Zhang, Ganghong Chen, Wei Zhou, Dazhi Yang

**Affiliations:** ^1^Department of Orthopedics, Huazhong University of Science and Technology Union Shenzhen Hospital, Shenzhen 518000, China; ^2^Department of Ultrasonics, Huazhong University of Science and Technology Union Shenzhen Hospital, Shenzhen 518000, China; ^3^Department of Orthopedics, Liyuan Hospital, Tongji Medical College, Huazhong University of Science and Technology, Wuhan 430077, China; ^4^Department of Orthopedics, The 6th Affiliated Hospital of Shenzhen University Health Science Center, Shenzhen 518000, China

## Abstract

DHEA-Box Helicase 37 (DHX37) is a putative RNA helicase. It is involved in various RNA secondary structure alteration processes, including translation, nuclear splicing, and ribosome assembly. It is reported to be associated with the neurodevelopmental disorder with brain anomalies, and a recent study suggests that DHX37 is a functional regulator of CD8 T cells. Dysregulation of the CD8 T cell function is closely related to defective antitumor immune responses. In the present study, we investigated the expression, mutation, and prognostic role of DHX37 in human cancers, mainly by mining publicly available datasets. Our results suggested that DHX37 was significantly upregulated in 17 kinds of tumors. Mutations including deletions, insertions, and substitutions of DHX37 were widely detected. Besides, the expression of DHX37 was negatively correlated with immune-related genes PD-L1, RGS16, and TOX, and it was positively associated with TIM3, LAG3, and NCOR2. Through biofunctional analysis, we observed that DHX37 was significantly enriched in cancer-related pathways such as cell cycle, DNA replication, mismatch repair, RNA degradation, and RNA polymerase. In conclusion, the study explored the significance of DHX37 in human cancers. DHX37 may serve as a potential target for cancer immunotherapy.

## 1. Introduction

With the rapid development of high-throughput sequencing technology, vast amounts of data carrying genetic information become publicly available. The Cancer Genome Atlas (TCGA) database provides data on the gene expression, mutation, methylation, and copy number variation in more than 18 million cancer cases. Recent studies devoted their efforts to developing bioinformatic methodologies to extract new information from the TCGA database. For example, prognostic signatures based on the gene expression or ncRNA expression were constructed in various kinds of cancers using data in the TCGA database. These signatures provide new biomarkers or therapeutic targets for cancer patients [1–4].

Human cancers have now become one of the leading causes of death worldwide. According to data from the International Agency for Research on Cancer, 9.6 million individuals died from cancer in 2018 [5]. The course of cancer initiation and development is still obscure, and great efforts are needed to explore mechanisms of carcinogenesis and develop potential therapeutic targets. Traditional treatment for cancers mainly includes surgery, chemotherapy, and radiotherapy. In recent years, immunotherapies such as immune checkpoint blockade and chimeric antigen receptor T cell therapy achieved great success in clinical trials [6–9]. However, the ability of cancer cells to escape from immunosurveillance limited the efficacy of immunotherapy [10]. Therefore, it is vital to reveal the mechanisms about how cancer cells evade the detection and elimination of the host immune system.

DHX37 is a member of the DExD/H-box RNA helicase family. Proteins in this family are evolutionarily conserved and are involved in modifying RNA secondary structures. A recent study suggested that DHX37 is highly expressed in human activated CD4+/CD8+ cells. Tumor Immune Dysfunction and Exclusion (TIDE) analysis indicated that the high expression of DHX37 is associated with worse prognosis in breast cancer [11]. However, very little is known about the expression, mutation, and prognostic role of DHX37 in human cancers.

In the present study, by utilizing data in public datasets, we compared the expression of DHX37 between normal tissues and tumor tissues. We found that DHX37 was significantly upregulated in 17/23 tumors. Various mutations of DHX37 were detected in human cancers. In addition, we investigated that the expression of DHX37 was correlated with several tumor-related immune genes (PD-L1, TIM3, LAG3, TOX, RGS16, and NCOR2), indicating that it may play crucial roles in tumor immune dysregulation. For survival analysis, DHX37 showed diverse prognostic values in different types of cancers. In conclusion, our study elucidated the potential role of DHX37 in cancers, and it may serve as a biomarker in some tumors. Further studies are still needed to verify our findings.

## 2. Materials and Methods

### 2.1. UALCAN Database

The UALCAN database (http://ualcan.path.uab.edu/) is a comprehensive, user-friendly, and interactive website tool for analyzing the cancer OMICS data [12]. The database can integrate gene expression data and clinical information of cancer patients in the TCGA database to provide gene expression graphs and plots in different patient groups. In the present study, we used the UALCAN database to compare the DHX37 expression between normal tissues and tumor tissues in 23 cancers from the TCGA database. *P* < 0.05 was considered significant.

### 2.2. Human Protein Atlas

We utilized data in the Human Protein Atlas to observe the DHX37 protein expression in human cancers (https://www.proteinatlas.org/). Human Protein Atlas is a website tool aiming to map all human proteins in cells, tissues, and organs by integrating various omic technologies [13]. In the present study, we checked the DHX37 protein expression in different cancer cells.

### 2.3. COSMIC and cBioPortal Database

The Catalog Of Somatic Mutations In Cancer (COSMIC) (https://cancer.sanger.ac.uk/cosmic) database is currently the broadest database for analyzing the impact of somatic mutations on human cancers. Over 4 million coding mutations, 13 million noncoding mutations, 18 thousand gene fusions, and 180 thousand genome rearrangements were described in the COSMIC database [14]. In this study, the COSMIC database was used for identifying mutations of DHX37 in human cancers. The cBioPortal (https://www.cbioportal.org/) database provides analysis and visualization of large-scale genomic datasets. To visualize DHX37 mutation status in human cancers, we carried out cBioPortal analysis, and the results were shown in bar plots.

### 2.4. Genome-Wide Association Analysis of DHX37

To identify genes that correlate with DHX37 and depict circus plots about the expression of DHX37 and these genes, we utilized the Multi-Scale Association Explorer (MSAE) tool on the Cancer Regulome website (http://www.cancerregulome.org/). Genes with −log_10_(*P*) ≥ 6 were regarded as significant genes and were shown in the circus plots.

### 2.5. Kaplan-Meier Curve for Survival Analysis

To investigate the prognostic role of DHX37 in human cancers, we used a website tool called The Kaplan-Meier Plotter (https://kmplot.com/analysis/) to draw the Kaplan-Meier curve. The website collects gene chip or RNA-seq data from 11,000 samples from 20 different cancer types. In the present study, we utilized the TCGA datasets to create survival curves for DHX37.

### 2.6. UCSC Xena for Gene Correlation Analysis

The UCSC Xena website tool was designed to analyze and visualize cancer genomic data from public and researchers' private datasets. As a previous study which demonstrated that DHX37 was associated with the CD8 T cell function, we analyzed the correlation between the expression of DHX37 and immune-related genes PD-L1 (CD274), HAVCR2 (TIM3), LAG3, TOX, RGS16, and NCOR2. Heatmaps were created using the Genes viewing mode.

### 2.7. KEGG and Metascape Enrichment Analysis

Biofunctional analysis of DHX37 was conducted. KEGG, GO, Reactome, and CORUM analysis were conducted using Metascape (http://metascape.org). Bubble plot was depicted by *R* Studio (Version 1.2.5033, ggplot2 package).

## 3. Results

### 3.1. The Expression of DHX37 Differs between Normal and Tumor Tissues in a Variety of Human Cancers

**Figure 1:**
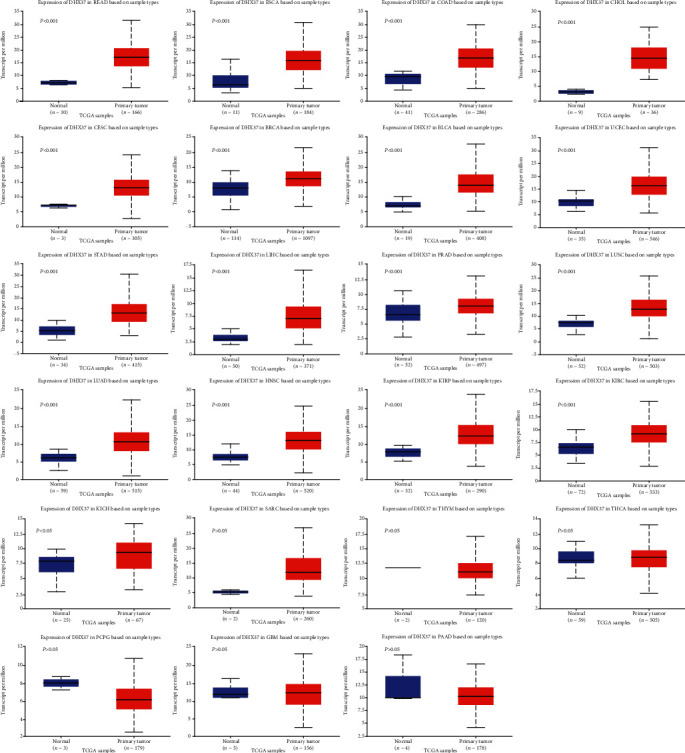
The expression of DHX37 in human cancers was investigated using the UALCAN database.

**Figure 2:**
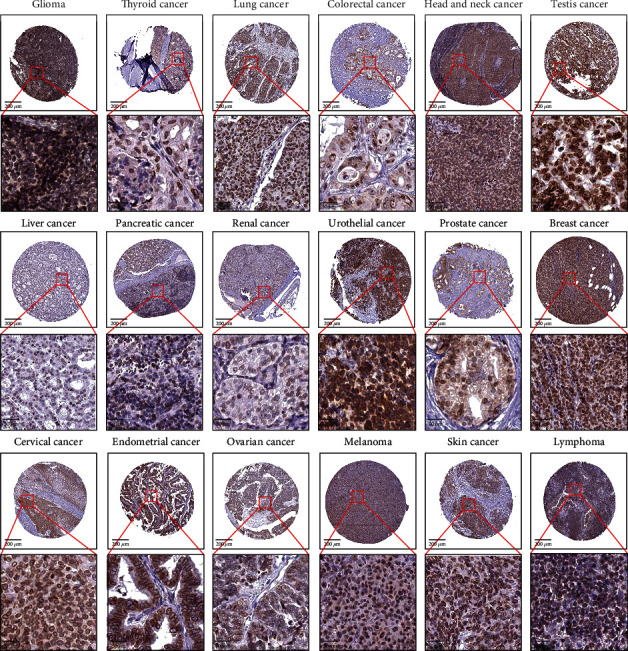
The protein expression of DHX37 in human cancer cells was evaluated using the Human Protein Atlas database.

Using the UALCAN website, we compared the expression of DHX37 between normal samples and tumor samples in 23 human cancers. DHX37 was significantly upregulated in 17 tumor tissues, including rectum adenocarcinoma (READ), esophageal carcinoma (ESCA), colon adenocarcinoma (COAD), cholangiocarcinoma (CHOL), cervical squamous cell carcinoma (CESC), breast invasive carcinoma (BRCA), bladder urothelial carcinoma (BLCA), uterine corpus endometrial carcinoma (UCEC), stomach adenocarcinoma (STAD), liver hepatocellular carcinoma (LIHC), prostate adenocarcinoma (PRAD), lung squamous cell carcinoma (LUSC), lung adenocarcinoma (LUAD), head and neck squamous cell carcinoma (HNSC), kidney renal papillary cell carcinoma (KIRP), kidney renal clear cell carcinoma (KIRC), and kidney chromophobe (KICH) compared to that in corresponding normal tissues (*P* < 0.05). No significant differences between normal and tumor tissues were observed in sarcoma (SARC), thymoma (THYM), thyroid carcinoma (THCA), pheochromocytoma and paraganglioma (PCPG), glioblastoma multiforme (GBM), and pancreatic adenocarcinoma (PAAD) (Figure 1). In addition, we also observed the protein expression of DHX37 in human cancer cells through the Human Protein Atlas database. The results were shown in Figure 2.

### 3.2. DHX37 Mutations in Human Cancers

**Figure 3:**
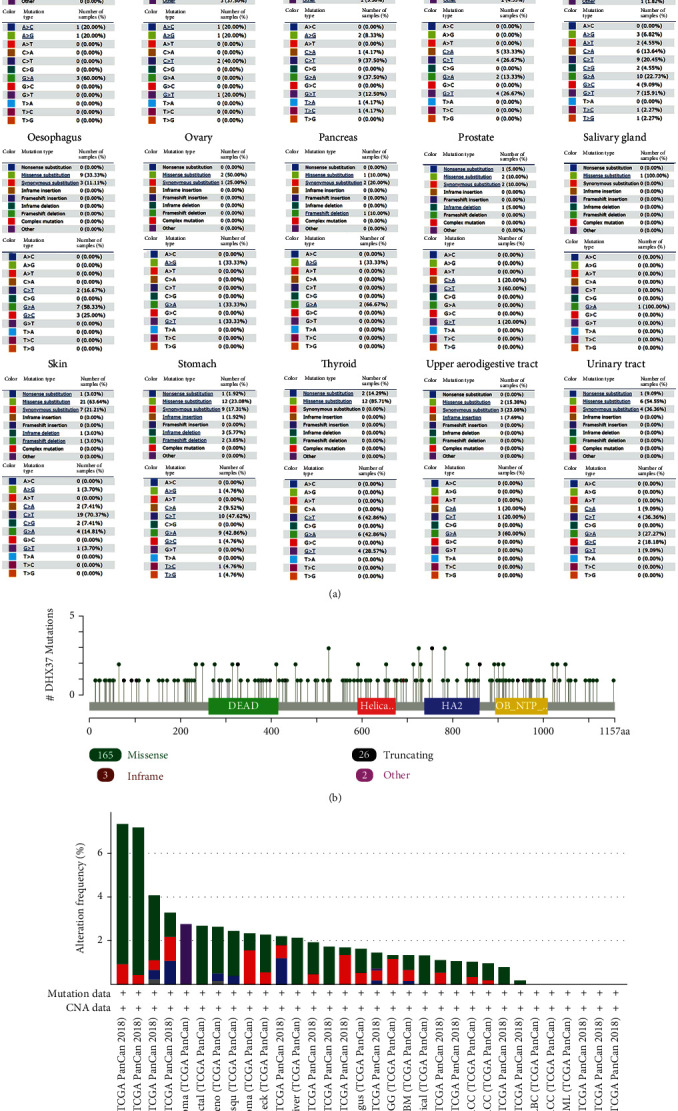
DHX37 mutations in human cancers. (a) The percentage of different mutation types of DHX37 in human cancers according to the COSMIC database. (b) Mutations of DHX37 in protein domains. (c) Mutation level of DHX37 using data from cBioPortal.

The data of DHX37 somatic mutations was obtained from the COSMIC database (Figure 3(a)). In human cancers, nonsense, missense, and synonymous substitution were major types of DHX37 somatic mutations. Nonsense substitutions were mainly found in central nervous system carcinoma (22.22%), endometrioid carcinoma (10%), large intestine carcinoma (2.78%), liver carcinoma (4.35%), lung carcinoma (1.82%), prostate carcinoma (5%), skin carcinoma (3.03%), stomach carcinoma (1.92%), thyroid carcinoma (14.29%), and urinary tract carcinoma (9.09%). According to different cancer types, missense substitutions were observed in all cancer types, with a percentage of 10%-100%. Cancers with over 50% missense substitutions were cervix carcinoma (50%), large intestine carcinoma (52.78%), ovary carcinoma (50%), salivary gland carcinoma (100%), skin carcinoma (63.64%), thyroid carcinoma (85.71%), and urinary tract carcinoma (54.55%). Synonymous substitutions occurred in many kinds of cancers except salivary gland cancer and thyroid cancer. The percentage of synonymous substitutions in different cancers was biliary tract carcinoma (23.08%), breast carcinoma (5.26%), central nervous system carcinoma (77.78%), cervix carcinoma(25%), endometrioid carcinoma (20%), hematopoietic and lymphoid carcinoma (14.29%), kidney carcinoma (25%), large intestine carcinoma (13.89%), liver carcinoma (10.87%), lung carcinoma (32.73%), esophagus carcinoma (11.11%), ovary carcinoma (25%), pancreas carcinoma (20%), prostate carcinoma (10%), skin carcinoma (21.21%), stomach carcinoma (17.31%), upper aerodigestive tract carcinoma (23.08%), and urinary tract carcinoma (36.36%), respectively. Additionally, inframe insertion was observed in stomach carcinoma (1.92%) and upper aerodigestive tract carcinoma (7.69%). Inframe deletion was observed in endometrioid carcinoma (10%), hematopoietic and lymphoid carcinoma (14.29%), liver carcinoma (2.17%), prostate carcinoma (5%), skin carcinoma (3.03%), and stomach carcinoma (5.77%). Frameshift deletion was found in large intestine carcinoma (2.78%), pancreas carcinoma (10%), skin carcinoma (3.03%), and stomach carcinoma (3.85%). No frameshift insertion of DHX37 was found in any cancer. A > C mutation was found in hematopoietic and lymphoid carcinoma (20%) and kidney carcinoma (20%). A > T mutation was observed in biliary tract carcinoma (14.29%), endometrioid carcinoma (7.69%), and lung carcinoma (4.55%). C > G mutation was seen in breast carcinoma (10%) and large intestine carcinoma (7.41%). T > A was seen in breast carcinoma (10%) and large intestine carcinoma (4.17%). T > C was observed in large intestine carcinoma (4.17%), lung carcinoma (2.27%), and stomach carcinoma (4.76%). T > G was seen in lung carcinoma (2.27%) and stomach carcinoma (4.76%). As shown in Figure 3(b), cBioPortal analysis suggested that 165 mutation points were detected in a total of 1157 amino acids of DHX37. The percentage of different mutation types in different cancers was shown in Figure 3(c).

### 3.3. Genome-Wide Association Analysis of DHX37 in Human Cancers

**Figure 4:**
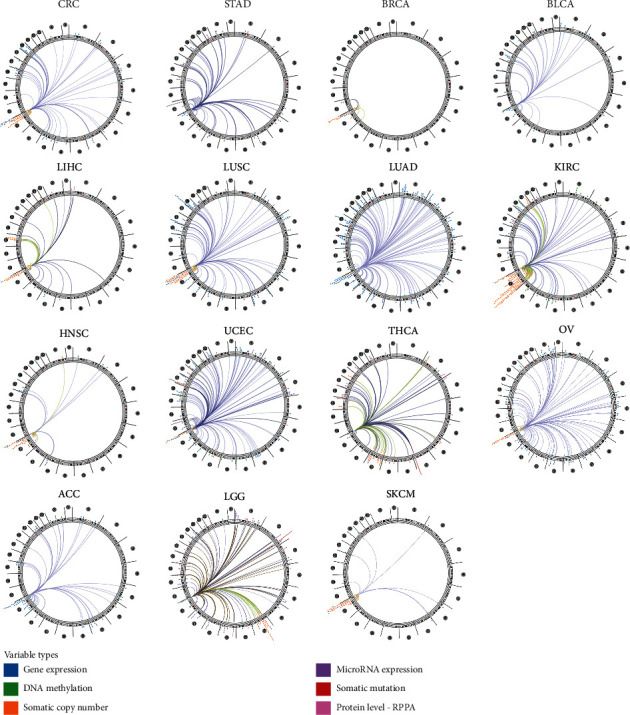
Correlation between DHX37 and other genes in different human cancers.

To obtain correlations between genes and DHX37 in a range of the whole genome, we carried out genome-wide association analysis. The results were mapped to the relevant human genome and were plotted in circus plots using Cancer Regulome (MASE). Mapping was based on the association among genes, copy number variants, microRNAs, and other genetic features. As shown in Figure 4, DHX37 was associated with genes that could be detected in colorectal cancer (CRC), STAD, BRCA, BLCA, LIHC, LUSC, LUAD, KIRC, HNSC, UCEC, THCA, ovarian serous cystadenocarcinoma (OV), adrenocortical carcinoma (ACC), brain lower grade glioma (LGG), and skin cutaneous melanoma (SKCM). Detailed information was listed in Supplementary 1.

### 3.4. Prognostic Role of DHX37 in Human Cancers

**Figure 5:**
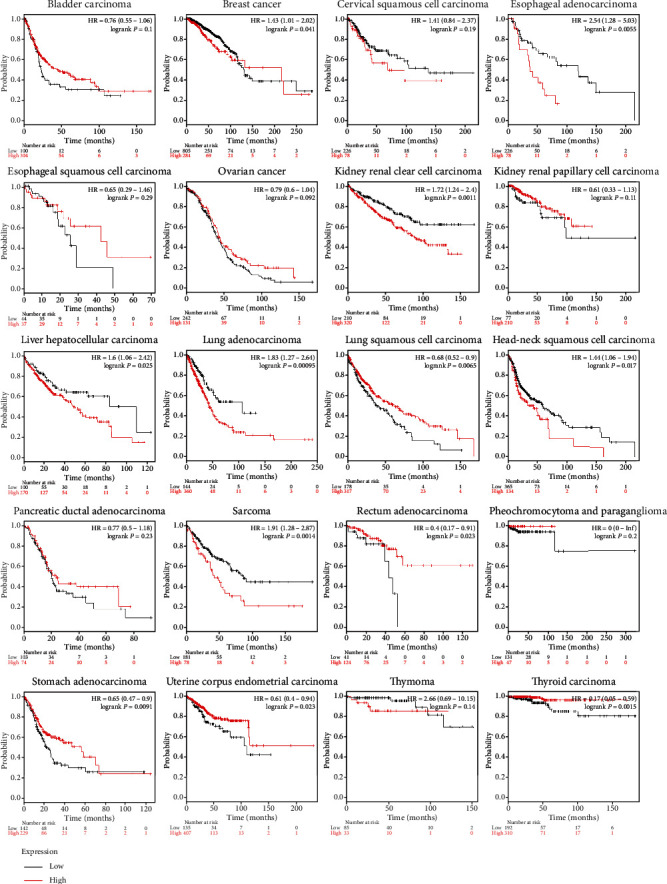
Kaplan-Meier curve showed that DHX37 has different effects on prognosis in different tumors.

To investigate the prognostic value of DHX37 in human cancers, we used the Kaplan-Meier Plotter to draw the survival curves. Patients were divided into a high-risk group and a low-risk group according to the website's median DHX37 expression. As shown in Figure 5, the DHX37 expression played different prognostic roles in human cancers. The high expression of DHX37 was significantly associated with unfavorable prognosis in esophageal adenocarcinoma (*P* = 0.0055), kidney renal clear cell carcinoma (*P* = 0.0011), liver hepatocellular carcinoma (*P* = 0.025), lung adenocarcinoma (*P* = 0.00095), head-neck squamous cell carcinoma (*P* = 0.017), and sarcoma(*P* = 0.0014). However, the opposite results were observed in lung squamous cell carcinoma (*P* = 0.0065), rectum adenocarcinoma (*P* = 0.0023), stomach adenocarcinoma (*P* = 0.0091), uterine corpus endometrial carcinoma (*P* = 0.023), and thyroid carcinoma(*P* = 0.0015), in which the high expression of DHX37 was correlated with favorable prognosis. Besides, in bladder carcinoma, cervical squamous cell carcinoma, esophageal squamous cell carcinoma, ovarian cancer, kidney renal papillary cell carcinoma, pancreatic ductal adenocarcinoma, pheochromocytoma, paraganglioma, and thymoma, DHX37 had no association with prognosis.

### 3.5. Correlation between DHX37 and Immune-Related Genes

**Figure 6:**
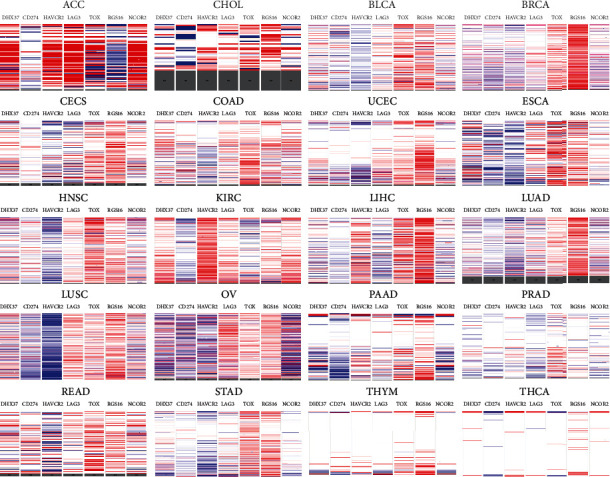
Gene expression relationship between DHX37 and CD274, HAVCR2, LAG3, TOX, RGS16, and NCOR2.

As DHX37 was involved in the regulation of CD8 T cell function, we next checked whether its expression was associated with the expression of some cancer-related immune genes by UCSC Xena. According to the data in TCGA tumor samples, the copy numbers of DHX37 were positively correlated with TIM3, LAG3 and NCOR2, while they were negatively correlated with PD-L1, RGS16 and TOX (Figure 6).

### 3.6. DHX37 Is Involved in Cancer Signaling Pathways

**Figure 7:**
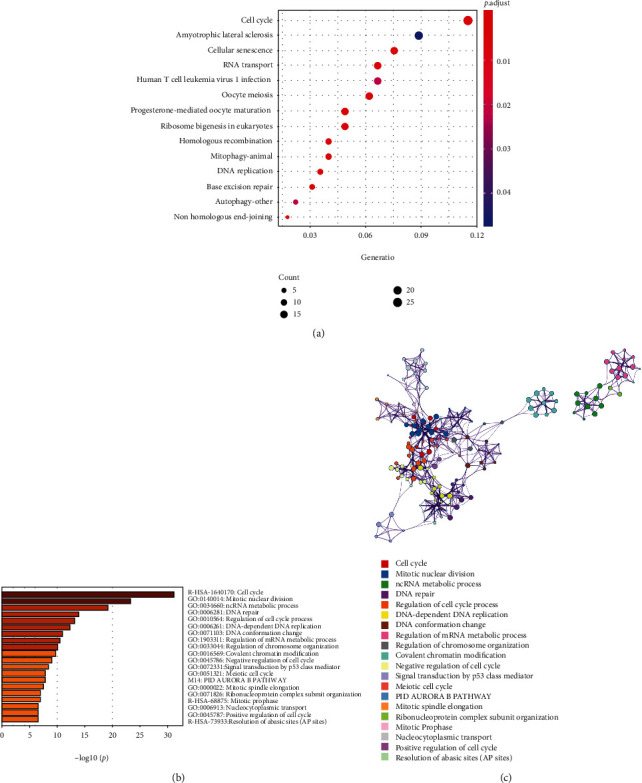
Pathway enrichment results of DHX37 in human cancers. (a) KEGG pathway analysis of genes associated with DHX37. (b).The bar graph and (c). The network visualization of pathway enrichment analysis using Metascape.

To further validate the function of DHX37 in human cancers, we conducted biofunctional analysis. KEGG analysis was performed using DHX37-related genes obtained from genome-wide association analysis. As shown in Figure 7(a), enriched signaling pathways related to DHX37 were cell cycle, homologous recombination, cellular senescence, DNA replication, and base excision repair et al. The results indicated that DHX37 was highly involved in cancer signaling pathways and may play critical roles in human cancer development. In addition, we carried out enrichment analysis based on all GO terms, KEGG pathways, Reactome, and CORUM using Metascape. The results were shown as the bar graph (Figure 7(b)) and the network visualization graph (Figure 7(c)).

## 4. Discussion

DHX37 is a member of the DExD/H-box RNA helicase family. Proteins in this family are revolutionary conserved, and they have either DExD or DExH amino acid sequence in their helicase core domain. These proteins' core domains participate in RNA and NTP binding, hydrolysis, substrate recognition, and helicase activity [15]. Although DExD/H box RNA helicases are widely expressed in human tissues, DHX37 shows the tissue-specific expression. DHX37 is expressed in most human organs, with the highest expression in lymphoid tissues, including bone marrow, spleen, lymph nodes, and appendix [16]. The function of DHX37 has not been well studied yet. Previous studies showed that DHX37 is specifically expressed in somatic cells of developing human testis, and pathogenic variants of DHX37 are a frequent cause of nonsyndromic 46, XY gonadal dysgenesis [17–19].

Few studies focused on the role of DHX37 in the human immune and cancers. Matthew et al. reported that in human breast cancer, DHX37 is expressed in both normal and tumor-associated T cells. Interactions between DHX37 and NF-*κ*B core components result in T cell dysfunction [11]. In addition, the high expression of DHX37 is found in both activated CD8+ and CD4 T+ cells, and the expression of DHX37 is significantly higher in exhausted tumor infiltration lymphocyte (TILs) compared to nonexhausted ones. These results suggested that the increased expression of DHX37 might be involved in tumor cell evasion from immune recognition.

In the present study, we found that the expression of DHX37 was significantly upregulated in 17 of 23 human cancers, including invasive breast cancer. The result is consistent with previous findings that DHX37 upregulation is associated with poor prognosis in breast cancer [11].

Next, we investigated the somatic mutations of DHX37 in human cancers. Mutations were widely observed. Among all the mutations, missense substitution was the most frequently occurred mutation and was found in all tumor types. Salivary gland carcinoma had 100% missense substitution, and thyroid carcinoma had 85.71%, suggesting that abnormal amino acid sequence of DHX37 may account for tumor development of these two cancers. The synonymous substitution was the second most common somatic mutation of DHX37. Besides, nonsense mutation, inframe insertion, inframe deletion, and frameshift deletion were observed in various tumor types, indicating that structural and functional changes of DHX37 are vital to tumor initiation and progression.

The relationship between DHX37 and tumor-related immune genes was also investigated. Our results suggested that the expression of DHX37 was positively correlated with TIM3, LAG3, and NCOR2, while it was negatively correlated with PD-L1, RSG16, and TOX. T cell immunoglobulin and mucin domain-containing protein 3 (TIM3) is expressed as the most on dysfunctional tumor-infiltrating CD8 + PD1+ T cells in cancer [20–22]. Blocking TIM3 can restore CD8 T cell responses to antitumor immunity [22]. Lymphocyte activation gene-3 (LAG3, CD223) is an inhibitory receptor. The upregulation of LAG3 is essential for limiting T cell activation and preventing the start of tumor immune responses in cancers [23]. As TIM3 and LAG3 are both potential targets for cancer immunotherapy, the clinical trial about coinhibition of TIM3 and LAG3 for cancer treatment is currently investigated [24]. These findings support our hypothesis that the high expression of DHX37 indicates an increased risk of tumor immune suppression and tumor progression. DHX37 may serve as a targetable inhibitory receptor for immunotherapy. PD-L1 is highly expressed in many tumors and is an inducer of T cell exhaustion [25]. PD-L1 is reasonable for cancer immune escape because it can weaken the host immune responses towards tumor cells [26]. The PD-1/PD-L1 axis is currently the most well studied inhibitory checkpoint molecules. The axis can be modulated by various signaling pathways, including PI3K/AKT, JAK/STAT3, WNT, NF-*κ*B, MAPK, and Hh pathway [26]. Targeting PD-1/PD-L1 is proved to be effective in treating both solid and hematological malignancies such as lung cancer [27], head and neck cancers [28], and lymphoma [25]. TOX is highly expressed in dysfunctional tumor-specific CD8 T cells. Knockdown of TOX can abrogate T cell exhaustion [29]. Reverse correlation between the expression of DHX37 and TOX or PD-L1 indicated that targeting DHX37 may be an alternative option for patients who are not sensitive to TOX or PD-L1 immunotherapy.

As far as we know, our study was the first to investigate the prognostic role of DHX37 in human cancers. Interestingly, the high expression of DHX37 was associated with favorable prognosis in lung squamous cell carcinoma, rectum adenocarcinoma, stomach adenocarcinoma, uterine corpus endometrial carcinoma, and thyroid carcinoma, which seemed to be not consistent with its role in cancer immune. This may because DHX37 was in the complicated regulatory networks of human cancers, and Figure 4 provides information about these gene-gene interactions.

In conclusion, our study is the first to investigate the expression, mutation, and prognostic role of DHX37 in human cancers. Our results suggest that DHX37 is upregulated in most human cancers, and it has different prognostic values in various cancers. Somatic mutations of DHX37 are widely found in cancers. In addition, the expression of DHX37 is correlated with critical inhibitory receptors in cancers. Biofunctional analysis shows that DHX37 is highly enriched in critical cancer signaling pathways. From the all above, DHX37 might serve as a potential target for cancer immunotherapy.

## Data Availability

The datasets generated during and/or analysed during the current study are available in the TCGA (http://cancergenome.nih.gov/), UALCAN (http://ualcan.path.uab.edu/), and COSMIC database (https://cancer.sanger.ac.uk/cosmic).

## Supplementary Materials

Supplementary Materials Detailed information of the gene expression in various cancers that associated with DHX37 were listed in Supplementary 1, which will be available online.
